# Vitamin B6 Prevents Endothelial Dysfunction, Insulin Resistance, and Hepatic Lipid Accumulation in* Apoe* ^−/−^ Mice Fed with High-Fat Diet

**DOI:** 10.1155/2016/1748065

**Published:** 2015-12-31

**Authors:** Zhan Liu, Peng Li, Zhi-Hong Zhao, Yu Zhang, Zhi-Min Ma, Shuang-Xi Wang

**Affiliations:** ^1^Department of Clinical Nutrition and Gastroenterology, The First Affiliated Hospital (People's Hospital of Hunan Province), Hunan Normal University, Changsha 430070, China; ^2^College of Pharmacy, Xinxiang Medical University, Xinxiang 453003, China; ^3^Division of Endocrinology, The Second Affiliated Hospital, Soochow University, Suzhou 215000, China; ^4^The Key Laboratory of Cardiovascular Remodeling and Function Research, Chinese Ministry of Education and Chinese Ministry of Health, Qilu Hospital, School of Medicine, Shandong University, Jinan 250012, China

## Abstract

*Backgrounds.* VitB6 deficiency has been associated with a number of adverse health effects. However, the effects of VitB6 in metabolic syndrome are poorly understood.* Methods.* VitB6 (50 mg/kg/day) was given to *Apoe* ^−/−^ mice with hkdigh-fat diet (HFD) for 8 weeks. Endothelial dysfunction, insulin resistance, and hepatic lipid contents were determined.* Results.* VitB6 administration remarkably increased acetylcholine-induced endothelium-dependent relaxation and decreased random blood glucose level in *Apoe* ^−/−^ mice fed with HFD. In addition, VitB6 improved the tolerance of glucose and insulin, normalized the histopathology of liver, and reduced hepatic lipid accumulation but did not affect the liver functions. Clinical and biochemical analysis indicated that the levels of VitB6 were decreased in patients with fatty liver.* Conclusions.* Vitamin B6 prevents endothelial dysfunction, insulin resistance, and hepatic lipid accumulation in *Apoe* ^−/−^ mice fed with HFD. Supplementation of VitB6 should be considered to prevent metabolic syndrome.

## 1. Introduction

Vitamin B6 (VitB6) includes pyridoxal, pyridoxine, and pyridoxamine, which function as essential cofactors for enzymes involved in various metabolic activities, which include amino acid, fat, and glucose metabolism [1]. The phosphate ester derivative pyridoxal 5′-phosphate (PLP) is the biologically active form of this vitamin and reflects long-term body storage [2]. Studies have shown that low plasma PLP concentrations are associated with increased risk of cardiovascular disease (CVD) [3, 4].

Nutrient overload is associated with high incidence of chronic metabolic diseases, including obesity, insulin resistance, and type 2 diabetes [5]. Prolonged exposure to high concentrations of saturated fatty acids leads to oxidative stress and endoplasmic reticulum stress, which may impair insulin signaling [6]. Moreover, supplementation of a high-fat diet (HFD) with branched-chain amino acids caused insulin resistance, as a part of metabolic syndrome [7]. Metabolic syndrome is associated with a risk of CVD and is a common early abnormality in the development of type 2 diabetes. In patients with nonalcoholic fatty liver disease (NAFLD), metabolic abnormalities have been reported in 33% to 100% of cases [8]. Patients presenting with NAFLD need to be examined for the presence of the components of the metabolic syndrome and their complications [9]. We also previously reported that apoptosis of liver cells contributes to liver dysfunction [10].

The identification of the link between VitB6 and metabolic syndrome including insulin and NAFLD might help to define novel nutritional and pharmacological approaches for the treatment of diabetes, obesity, and insulin resistance. Here, we reported that administration of VitB6 prevents endothelial dysfunction, insulin resistance, and hepatic lipid accumulation in *Apoe* ^−/−^ mice fed with high-fat diet. Clinically, deficiency of VitB6 should be considered as a high risk factor of NAFLD.

## 2. Materials and Methods

### 2.1. Materials

Human recombinant insulin was purchased from Sigma-Aldrich (St. Louis, MO). Antibodies against Akt (pAkt), GLUT4, glycogen synthase kinase-3*β* (GSK3), forkhead box protein O (FOXO), and GAPDH were purchased from Santa Cruz Biotechnology (Dallas, TX). The secondary antibodies were obtained from Jackson ImmunoResearch Laboratories (West Grove, PA). VitB6, acetylcholine (ACh), sodium nitroprusside (SNP), and phenylephrine were from Sigma-Aldrich Company. All drug concentrations are expressed as final working concentrations in the buffer.

### 2.2. Animals and Experimental Protocols

Male *Apoe* ^−/−^ mice were purchased from Hua-Fu-Kang Animal Company (Beijing, China). All animals were housed in temperature-controlled cages with a 12-hour light-dark cycle and given free access to water and normal chow. This study was carried out in strict accordance with the recommendations in the* Guide for the Care and Use of Laboratory Animals* of the National Institutes of Health.

Model of hyperlipidemia was induced by feeding mice with HFD containing 0.21% cholesterol and 21% fat (Research Diets Inc., D12079B). This diet was administered at 6 weeks of age and continued for 8 consecutive weeks. At 6 weeks of age, VitB6 (50 mg/kg/day) was also added to the drinking water for 8 weeks. The animal protocol was reviewed and approved by the Animal Care and Use Committee of Hunan Normal University.

### 2.3. Determinations of Serum Lipid Profiles and Liver Functions

Blood was sampled from mice for determination of total bilirubin (TB), aspartate aminotransferase (AST), alanine aminotransferase (ALT), alkaline phosphatase (ALP), and albumin (ALB). Serum levels of TB, AST, ALT, AP, and ALB were determined by commercial kits (Nanjing Jiancheng Biology Company, Nanjing, China).

### 2.4. Organ Chamber

*In vivo* or* ex vivo* organ chamber study was performed as described previously [11]. Mice were sacrificed under anesthesia by intravenous injection with pentobarbital sodium (30 mg/kg). The descending aorta isolated by removing the adhering perivascular tissue carefully was cut into rings (2-3 mm in length). Aortic rings were suspended and mounted to organ chamber by using two stainless hooks. The rings were placed in organ baths filled with Krebs buffer of the following compositions (in mM): NaCl, 118.3; KCl, 4.7; MgSO_4_, 0.6; KH_2_PO_4_, 1.2; CaCl_2_, 2.5; NaHCO_3_, 25.0; EDTA, 0.026; pH 7.4 at 37°C; and they were gassed with 95% O_2_ plus 5% CO_2_, under tension of 1.0 g, for 90-minute equilibration period. During this period, the Krebs solution was changed every 15 min. After the equilibration, aortic rings were challenged with 60 mM KCl. After washing and another 30-minute equilibration period, contractile response was elicited by phenylephrine (1 *μ*M). At the plateau of contraction, accumulative ACh (0.01, 0.03, 0.1, 0.3, 1, and 3 *μ*M) or SNP (0.01, 0.03, 0.1, 0.3, 1, 3, and 10 *μ*M) was added to induce the relaxation.

### 2.5. Glucose Tolerance Test (GTT) and Insulin Tolerance Test (ITT)

As described previously [12], glucose (2.0 g/kg) was given to mice (i.p.) after an overnight fast. Blood glucose (BG) levels were then measured at indicated times with a portable glucose meter (LifeScan, Milpitas, CA) after tail snipping. For ITT, mice were injected with insulin (0.55 IU/kg, i.p.) after 6-hour fast. BG levels were measured at indicated times with a portable glucose meter (LifeScan, Milpitas, CA) after tail snipping.

### 2.6. HE or Oil Red O Staining

Histological specimens were taken at the end of the study period for all mouse groups as described previously [13]. For each mouse, liver segments were fixed in 4% buffered formaldehyde and embedded in paraffin for histological analysis. Sections (5 *μ*m) were stained with either hematoxylin or eosin. Degree of severity of liver fibrosis was derived from blind analysis of each of the animals in each group. To determine hepatic lipid accumulation, frozen liver sections were stained with 0.5% Oil Red O for 10 min, washed, and counterstained with Mayer's hematoxylin for 45 sec. Data for Oil Red O staining were presented as the mean percentage of stained area to a total hepatic region in 10 fields from each liver section. Quantitative analysis was performed using analySIS-FIVE program (Olympus Soft Imaging System, Münster, Germany).

### 2.7. Western Blotting

The protocol for western blot was described as previously with some modifications [14]. Liver tissues were homogenized and the protein content in supernatant was assayed by BCA protein assay reagent (Pierce, USA). 20 *μ*g proteins were loaded to SDS-PAGE and then transferred to membrane. Membrane was incubated with 1 : 1000 dilution of primary antibody, followed by 1 : 2000 dilution of horseradish peroxides-conjugated secondary antibody. Protein bands were visualized by ECL (GE Healthcare, USA). The intensity (area × density) of the individual bands on western blots was measured by densitometry (model GS-700, Imaging Densitometer; Bio-Rad). The background was subtracted from the calculated area. The average of density for the bands in control group is considered as 100%.

### 2.8. Measurement of Cholesterol and Triglyceride Contents in Liver

Lipids in mouse liver were extracted as described by Folch et al. [15, 16]. Cholesterol and triglyceride levels in extracted lipids were measured enzymatically using the reagents from Cayman Chemical (Ann Arbor, MI) according to the manufacturer's instruction.

### 2.9. Statistical Analysis

The results were expressed as mean ± SEM. One-way ANOVA followed by *t*-test was used for two groups' comparison. *P* < 0.05 was considered significant.

## 3. Results

### 3.1. VitB6 Prevents Endothelial Dysfunction in* Apoe* ^−/−^ Mice Fed with HFD

Endothelial dysfunction has been identified as an early hallmark of CVD, such as atherosclerosis and hypertension [17–20]. We firstly determined whether VitB6 prevents endothelial dysfunction in mice with metabolic syndromes. The hyperlipidemia model was induced by feeding *Apoe* ^−/−^ mice with HFD [21]. As indicated in Table 1, HFD in *Apoe* ^−/−^ mice dramatically increased serum levels of triglyceride, cholesterol, and LDL, indicating that the model is successfully established. Importantly, the random level of blood sugar was also increased in *Apoe* ^−/−^ mice fed with HFD. However, treatment of these mice with VitB6 did not alter the levels of triglyceride, cholesterol, and LDL, except for random level of blood glucose.

**Figure 1:**
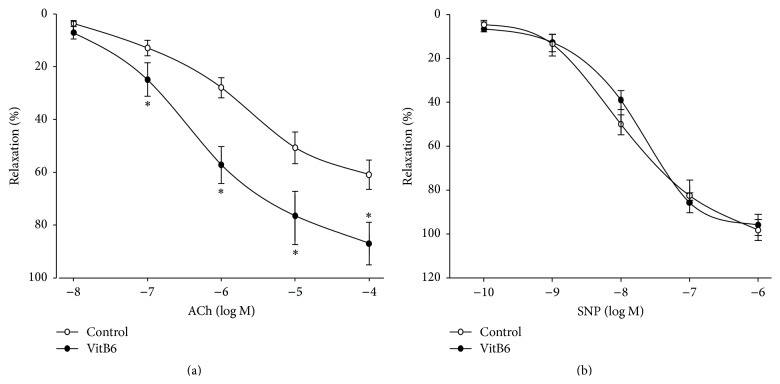
Administration of VitB6 prevents endothelial dysfunction in *Apoe* ^−/−^ mice fed with high-fat diet. Male *Apoe* ^−/−^ mice at the age of 6 weeks received high-fat diet and VitB6 (50 mg/kg/day) administration in drinking water for 8 weeks. At the end of experiments, mice were sacrificed under anaesthesia. The descending aortas were isolated and cut into rings. (a) ACh-induced endothelium-dependent relaxation and (b) SNP-induced endothelium-independent relaxation were determined by organ chamber as described in Section 2. All data were expressed as mean ± SEM. *N* is 10–15 in each group. ^*∗*^ *P* < 0.05 versus control.

The endothelial function was determined by using ACh. As shown in Figure 1(a), ACh-induced vasorelaxation was significantly improved by VitB6. The SNP-induced vasorelaxation was not affected by VitB6 (Figure 1(b)), demonstrating that the protective effects of VitB6 on vascular function are limited to endothelium.

### 3.2. VitB6 Enhances Insulin Sensitivity in* Apoe* ^−/−^ Mice Fed with HFD

**Figure 2:**
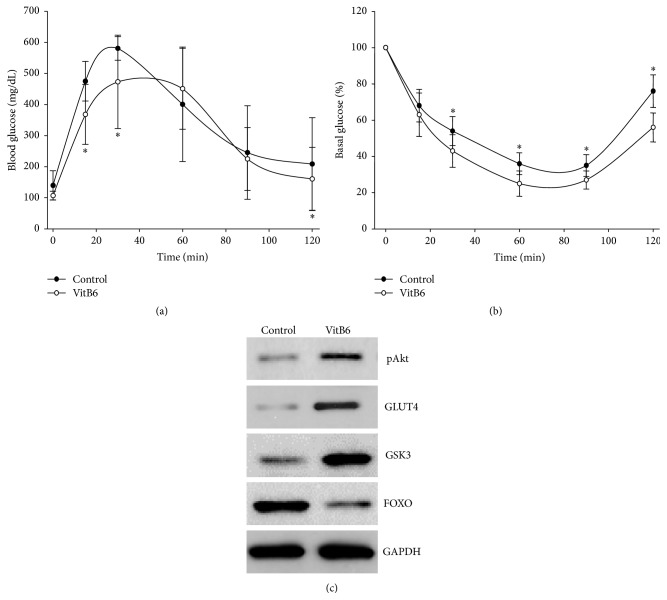
VitB6 improves insulin resistance in *Apoe* ^−/−^ mice fed with high-fat diet. Male *Apoe* ^−/−^ mice at the age of 6 weeks received high-fat diet and VitB6 (50 mg/kg/day) administration in drinking water. At the 8th weekend after VitB6 treatment, (a) GTT and (b) ITT were evaluated as described in Section 2. All data were expressed as mean ± SEM. *N* is 10–15 in each group. ^*∗*^ *P* < 0.05 versus control. (c) Homogenates of liver tissues were subjected to perform western blotting analysis to assay the levels of pAKt, GLUT4, GSK3, and FOXO. The picture is a representative blot from 10–15 mice.

Insulin resistance is a high risk factor of endothelial dysfunction in CVD [22]. We next examined whether VitB6 improves insulin sensitivity in HFD-fed *Apoe* ^−/−^ mice. As shown in Figure 2(a), injection of D-glucose dramatically increased the levels of blood glucose (BG) in HFD-fed *Apoe* ^−/−^ mice. The peak level of BG was about 600 mg/dL after 30 minutes. The level of BG was back to the basal level after 90 minutes. However, administration of VitB6 delayed and lowered the peak levels of BG (470 mg/dL). After 90 minutes of glucose injection, the level of BG was also back to the basal level. These data indicate that VitB6 increases the tolerance of glucose.

The protective effect of VitB6 on glucose metabolism was further confirmed by measuring the sensitivity of insulin (Figure 2(b)). By injecting exogenous insulin into HFD-fed *Apoe* ^−/−^ mice, the levels of BG were reduced to 40% of basal level at the 60th minute and then went back to 80% of basal level at the 120th minute. However, VitB6 further reduced the level of BG at the 90th minute to 25%. After the 120th minute, the level of BG was 55% of the basal level. Collectively, this suggests that VitB6 enhances insulin sensitivity in mice.

### 3.3. Increased Hepatic Levels of pAkt, GSK3, and GLUT4 Proteins and Decreased FOXO Protein Expression in VitB6-Treated* Apoe* ^−/−^ Mice

The beneficial effects of VitB6 on insulin resistance were further examined by assaying the hepatic levels of pAkt, GSK3, GLUT4, and FOXO, which are proteins related to glucose metabolism [23]. As depicted in Figure 2(c), compared to HFD-fed *Apoe* ^−/−^ mice, the levels of pAkt, GSK3, GLUT4, and BG were increased and the level of FOXO was reduced in HFD-fed *Apoe* ^−/−^ mice with VitB6, further supporting the notion that VitB6 improves insulin resistance in mice.

### 3.4. VitB6 Treatment Prevents Hepatic Lipid Accumulation in Mice

**Figure 3:**
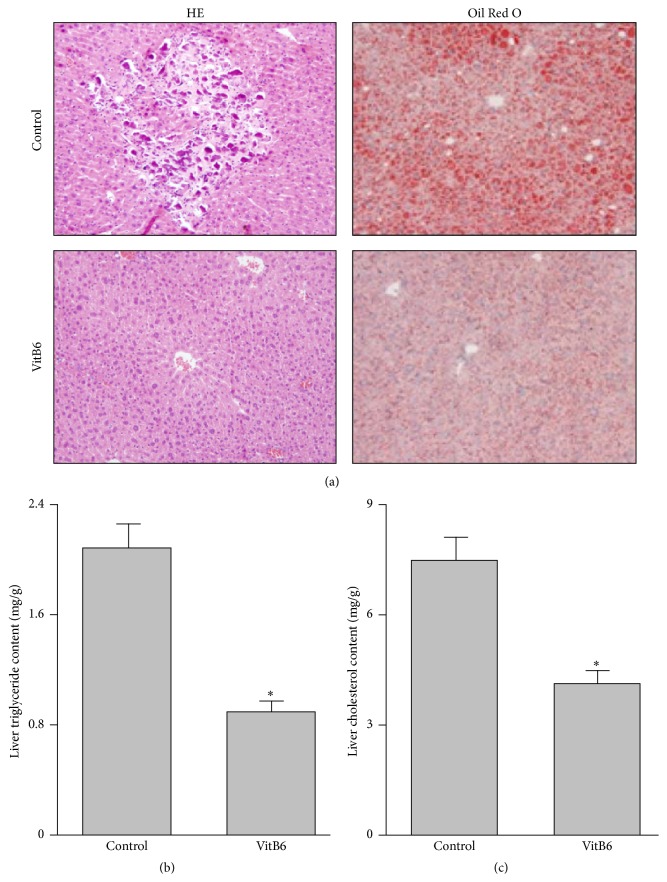
VitB6 reduces hepatic lipid accumulation in *Apoe* ^−/−^ mice fed with high-fat diet. Male *Apoe* ^−/−^ mice at the age of 6 weeks received high-fat diet and VitB6 (50 mg/kg/day) administration in drinking water. At the end of experiments, mice were sacrificed under anaesthesia. (a) Histological analysis of liver tissue by HE or Oil Red O staining. (b and c) Liver lipids were extracted and hepatic triglyceride and cholesterol levels were assayed using a commercial kit. The quantitative data were expressed as mean ± SEM. *N* is 10–15 in each group. ^*∗*^ *P* < 0.05 versus Control.

NAFLD is characterized by insulin resistance [24]. Thus, we detected the liver function in these mice. In Table 2, the markers of liver function, such as ALB, ALP, ALT, AST, and TB, were comparable in HFD-fed *Apoe* ^−/−^ mice with or without VitB6 treatment. Histological analysis of HE staining in liver sections from mice at the end of the experiment (Figure 3(a)) revealed that HFD caused marked neurosis and fibrosis, which was reversed by VitB6 treatment, suggesting that VitB6 is effective to protect the liver.

The typical feature of NAFLD is the elevated hepatic lipid accumulation [25, 26]. We next investigated whether VitB6 prevents hepatic lipid accumulation in hyperlipidemia mice by Oil Red O staining (Figure 3(a)). Compared to control HFD-fed *Apoe* ^−/−^ mice, the contents of liver triglycerides (Figure 3(b)) and cholesterol (Figure 3(c)) were decreased, demonstrating that VitB6 prevents hepatic lipid accumulation in mice and is potentially considered to serve as prevention of NAFLD.

### 3.5. Plasmatic Lower Levels of VitB6 in Patients with Fatty Liver

Finally, in order to establish the clinical association between VitB6 deficiency and NAFLD, we performed clinical and biochemical analysis. As described in Table 3, fifty-seven healthy humans and forty-nine patients had a clinical and biochemical analysis completed in the study. Compared to the healthy human subjects, the levels of folic acid were similar in patients with fatty liver. The levels of VitB12 were lightly increased. However, the levels of homocysteine in NAFLD patients were significantly increased, consistent with other reports [27, 28]. Most importantly, we found that the levels of VitB6 were lower in NAFLD than control healthy humans. These results indicate that deficiency of VitB6 might be a risk factor of NAFLD clinically.

## 4. Discussion

In the present study, we provide the first evidence that administration of VitB6 prevents endothelial dysfunction, insulin resistance, and hepatic lipid accumulation in *Apoe* ^−/−^ mice fed with HFD* in vivo*. Clinically, the serum level of VitB6 is low in patients with NAFLD. Our data not only indicate that VitB6 protects endothelial function and improves insulin resistance, but also imply that low VitB6 status might be a risk factor of NAFLD, as a component of metabolic syndrome.

The major discovery in the present study is that VitB6 produces several beneficial effects to prevent metabolic syndrome, such as insulin resistance and NAFLD. Traditionally, VitB6, in the form of PLP, is the coenzyme of 5 enzymes in these metabolic pathways: cystathionine-*β*-synthase (CBS), cystathionine-*γ*-lyase (CGL), cytoplasmic and mitochondrial serine hydroxymethyltransferase (cSHMT and mSHMT), and glycine decarboxylase (GDC) in the mitochondria [29]. In this way, VitB6 regulates the transsulfuration pathway which contributes to homocysteine regulation and provides cysteine synthesis and consists of sequential reactions catalyzed by CBS and CGL. CBS catalyzes the condensation of homocysteine and serine to form cystathionine in a reaction that is subject to positive allosteric regulation by S-adenosylmethionine (SAM), whereas CGL catalyzes the cleavage of cystathionine to yield *α*-ketobutyrate, ammonia, and cysteine. Because both CBS and CGL require PLP as a coenzyme, inadequate VitB6 status might lead to impaired regulation of cellular homocysteine concentration. High levels of homocysteine impair endothelial function and cause metabolic syndrome including insulin resistance and lipid accumulation in liver. HHCY might play a role in the pathogenesis of vascular disorders and is considered as an independent risk factor for atherosclerosis [30]. From our observations, supplementation of VitB6 should be a helpful therapy to improve endothelial dysfunction and metabolic syndrome. Of course, the mechanism of VitB6 in prevention of metabolic syndrome needs further investigations.

We also identified VitB6 deficiency as a new risk factor of NAFLD. Obesity, metabolic syndrome, and type 2 diabetes mellitus are strictly related and are key pathogenetic factors of NAFLD, the most frequent liver disease worldwide. NAFLD is a clinicopathological syndrome including a wide spectrum of liver damage instances, ranging from hepatic steatosis to nonalcoholic steatohepatitis (NASH) to cirrhosis [31]. Epidemiologic studies showed that low VitB6 nutritional status is associated with increased risk of CVD, venous thrombosis, stroke, and possibly colon cancer [32]. Although a connection between VitB6 status and lipid metabolism has appeared periodically for more than 80 years, there is no evidence to support the role of PLP in NAFLD. To our knowledge, this is the first study to investigate whether marginal VitB6 deficiency affects hepatic lipid accumulation in human adults. We observed a significant decrease of plasma PLP concentration in patients with NAFLD. A potential mechanism responsible for the observations of lower plasma VitB6 level linking to NAFLD is impairment of PUFA interconversion because it has been reported that marginal VitB6 deficiency decreases plasma (n-3) and (n-6) PUFA concentrations in healthy men and women [33]. Further investigation should focus on the direct target of VitB6 on regulation of lipid metabolism in liver.

A limitation of this study is that *Apoe* ^−/−^ mouse is suitable for studying atherosclerosis resulting from hypercholesterolemia. Additionally, this mouse has several intriguing characteristics. First, *Apoe* ^−/−^ mice show obesity-resistant phenotype, resulting in remarkable insulin sensitivity. Second, this mouse has hepatic steatosis due to impairment of VLDL secretion from liver. Third, this mouse basically possesses endothelial dysfunction damaged from excess beta lipoprotein. It would be better to investigate the metabolic effects of vitamin B6 on wild-type mice with diet-induced metabolic disorders, such as C57B16 strain.

In summary, the results of this study have shown that low VitB6 status has substantial effects on metabolism including glucose and fatty acid. The results of this study also demonstrate that the deficiency of VitB6 might be a risk factor of NAFLD.

## Figures and Tables

**Table 1:** Serum sugar and lipid levels in *Apoe* ^−/−^ mice.

	WT	*Apoe* ^−/−^		
	ND	ND	HFD	HFD + VitB6
Glucose (mM)	6.5 ± 1.3	8.2 ± 1.3	13.5 ± 0.8	10.2 ± 0.9^*∗*^
Cholesterol (mM)	3.7 ± 0.5	10.5 ± 2.1	28.4 ± 5.3	26.4 ± 4.9
Triglyceride (mM)	0.7 ± 0.2	0.8 ± 0.2	1.6 ± 0.3	1.5 ± 0.5
HDL-C (mg/L)	128 ± 15	252 ± 30	267 ± 38	257 ± 39
LDL-C (mg/L)	109 ± 14	249 ± 23	417 ± 53	435 ± 67

After 8-week administration of VitB6 in *Apoe* ^−/−^ mice fed with high-fat diet, serum sugar levels and lipid levels were determined. WT: wild-type; ND: normal diet; HFD: high-fat diet; HDL: high density lipoprotein; LDL: low density lipoprotein. All data were expressed as mean ± SEM. *N* is 10–15 in each group. ^*∗*^ *P* < 0.05 versus *Apoe* ^−/−^ mice fed with HFD.

**Table 2:** The indexes for liver function in mice.

	Control	VitB6
AST (IU/L)	132.2 ± 27.9	157.4 ± 31.8
ALT (IU/L)	186.7 ± 21.3	195.7 ± 28.6
TB (mg/dL)	0.13 ± 0.07	0.15 ± 0.09
ALP (IU/L)	255.5 ± 32.8	279.8 ± 35.8
ALB (g/L)	17.2 ± 1.4	20.6 ± 16.8

After 8-week administration of VitB6 in *Apoe* ^−/−^ mice fed with high-fat diet, serum levels of AST, ALT, TB, ALP, and ALB were determined. All data were expressed as mean ± SEM. *N* is 10–15 in each group.

**Table 3:** The levels of serum VitB6, homocysteine, folate, and VitB12 in patients with fatty liver.

	Control (57)	Patients (49)
VitB6 (PLP, nM)	55.8 ± 10.7	23.9 ± 8.1^*∗*^
VitB12 (pg/mL)	686.7 ± 21.3	518.7 ± 28.6^*∗*^
folic acid (ng/mL)	8.3 ± 0.7	7.7 ± 0.9
Homocysteine (nM)	15.8 ± 2.8	22.8 ± 5.4^*∗*^

Serum levels of VitB6, VitB12, folic acid, and homocysteine were determined in patients with fatty liver and control subjects. All data were expressed as mean ± SEM. ^*∗*^ *P* < 0.05 versus control.
